# Supplementary material for: Constant-pH Simulation of the Human β2 Adrenergic Receptor Inactivation
Source: J Chem Inf Model. 2025 Sep 29;65(19):10634–45. doi: 10.1021/acs.jcim.5c01641 (PMC12529767; doi:10.1021/acs.jcim.5c01641)
Supplement: Supplementary file 1 [file ci5c01641_si_001.pdf]

Supporting Information for: "Constant-pH simulation of the human β_2 adrenergic receptor inactivation"

Federico Ballabio[†,‡] and Riccardo Capelli[*,†]

†*Department of Biosciences, Università degli Studi di Milano, Via Celoria 26, I-20133 Milano, Italy*

‡*Institute of Biophysics, National Research Council of Italy (IBF-CNR), Via Celoria 26, I-20133 Milano, Italy*

E-mail: riccardo.capelli@unimi.it

Description of the Na^+ binding pathways

We performed a systematic analysis of the MD trajectories using the MDAnalysis library (version 2.7),[1] focusing on the Na^+ ions which moved close to residues $D79^{2x50}$ and $D113^{3x32}$ (Figure S13).

In replica 2 at pH 7, a single ion was observed to reach a minimum distance from $D79^{2x50}$ of 8.1 Å; in replica 3 at pH 8, the minimum distance was 9.4 Å; in replica 5 at pH 8, the minimum distance was 9.9 Å; in replica 5 at pH 9, the minimum distance was 8.9 Å. Analysis of the trajectories showed that these minimum distances corresponded to interactions with residue $D113^{3x32}$. Regarding the latter residue $D113^{3x32}$, no sodium ions approached within 5 Å at pH 4 or pH 5. Interactions were observed from pH 6 onward.

Considering the sodium entrance in every simulation, we have that for pH 6, a single binding event lasting 16.4 ns occurred at the end of replica 1 (distance plot S13A). In replica

4, three sodium ions interacting with the residue were observed at different times for a total of 6.5 ns (distance plot S13B). Interestingly, the ion in replica 1 also interacted with residues E180^{ECL2} and D300^{ECL3} in the interval between ∼622.5 ns and ∼655.5 ns (distance plot A). At pH 7, residue D113^{3x32} is engaged by ions in all five replicas. In replica 1, the first binding event occurred at 333.6 ns and lasts 1.8 ns. A different sodium ion entered at 431.7 ns and remained for 84.9 ns (distance plot S13C). The most persistent event occurred in replica 2, where the ion remained within 5 Å of the residue for 826.6 ns (distance plot S13D). Similarly, replica 3 showed a highly stable interaction, with the ion maintaining contact for 482 ns (distance plot S13E), while replicas 4 and 5 displayed shorter contacts of 15.2 ns and 4.9 ns (distance plots S13F and S13G), respectively. At pH 8, differently from pH 7, the frequency of multiple independent binding events for a single replica increased: nine ion-residue interactions in replica 3 and ten in replica 5. In detail, replica 1 featured three ions contacting the residue from 82.6 to 484.0 ns (distance plot S13H). Replica 2 showed a single, highly stable event (the longest observed here), with the ion maintaining the interaction for 920.7 ns (distance plot S13I). Replica 3 displayed a broad diversity of transient and stable events, with interaction durations varying from 0.1 ns to 102.7 ns (distance plot S13J). Replica 4 highlighted another long event, with the ion bound to the residue for 431.3 ns (distance plot S13K). Replica 5 contributed additional interactions, ranging from 3.3 ns to 85.2 ns (distance plot S13L). At pH 9, interaction events with residue 3x32 persisted across all replicas. Notably, only replica 1 exhibited a single binding event, which is the second most stable in the dataset, lasting 859.4 ns (distance plot S13M). Replica 2 featured multiple ions interacting at staggered times (distance plot S13N). Replica 3 included a highly persistent contact of 739.4 ns (distance plot S13O), while replica 4 showed both transient and stable events, including a 101.8 ns contact (distance plot S13P). Finally, replica 5 was marked by a particularly long interaction with the ion that remains within 5 Å of residue D113^{3x32} for over 519.1 ns (distance plot S13Q).

According to the microswitches analysis (see the corresponding section in the main text),

the pH 7 systems showed a better agreement in the majority of the features considered indicative of receptor inactivation during the simulation. Based on this observation, we focused our attention on the trajectories at pH 7, paying particular attention to the paths taken by sodium ions towards residue D113^{3x32}. We analyzed the sequence of polar and negatively charged residues encountered along the trajectory of each ion that reached D113^{3x32} within 5 Å. These residues were defined as those interacting within 5 Å of the ion. Two independent ion binding events were identified in replica 1. During the first event, the ion first engaged with residue D300^{ECL3}, then moved through T195^{ECL2}, before being coordinated by both D113^{3x32} and N312^{7x38}. As previously described, this interaction was relatively short-lived, lasting around 1.8 ns. The second event in the same replica was notably longer. In this case, a different ion interacted with D192^{ECL2} before continuing through the extracellular cavity via T195^{ECL2}, and ultimately reached D113^{3x32} and N312^{7x38}. In replica 2, a single ion enters via D300^{ECL3} and Q197^{5x37}. It then proceeded through T195^{ECL2} to N293^{6x55} and S203^{5x43} before finally reaching D113^{3x32}. In Replica 3, the ion path involved initial contacts with E306^{7x32} and D192^{ECL2}, followed by coordination with N312^{7x38} before engaging with D113^{3x32}. Replica 4 presented a more complex trajectory. First, the ion interacted with D192^{ECL2}, then with Q197^{5x37}. Subsequently, it engaged with T195^{ECL2} before returning to D192^{ECL2} and D300^{ECL3}. Finally, it stabilized at D113^{3x32} assisted by N312^{7x38}. In replica 5, the ion entered through D300^{ECL3}, D192^{ECL2}, and moved to D113^{3x32} and N312^{7x38}, eventually contacting S203^{5x43}. These observations suggested that the approach of ions to D113^{3x32} involves consistent coordination by acidic and polar residues, such as D300^{ECL3}, D192^{ECL2}, and T195^{ECL2}.

Table S1: Ballesteros-Weinstein generic numbering for human β_2-adrenoceptor, as listed in the GPCRdb[2] (https://gpcrdb.org/residue/residuetabledisplay, accessed on December 2024).

**TM1**		**TM2**		**TM3**		**TM4**		**TM5**		**TM6**		**TM7**	
1x25	Q26	2x37	T66	3x21	G102	4x38	T146	5x36	N196	6x24	S262	7x30	R304
1x26	E27	2x38	V67	3x22	N103	4x39	K147	5x37	Q197	6x25	K263	7x31	K305
1x27	R28	2x39	T68	3x23	F104	4x40	N148	5x38	A198	6x26	F264	7x32	E306
1x28	D29	2x40	N69	3x24	W105	4x41	K149	5x39	Y199	6x27	C265	7x33	V307
1x29	E30	2x41	Y70	3x25	C106	4x42	A150	5x40	A200	6x28	L266	7x34	Y308
1x30	V31	2x42	F71	3x26	E107	4x43	R151	5x41	I201	6x29	K267	7x35	I309
1x31	W32	2x43	I72	3x27	F108	4x44	V152	5x42	A202	6x30	E268	7x36	L310
1x32	V33	2x44	T73	3x28	W109	4x45	I153	5x43	S203	6x31	H269	7x37	L311
1x33	V34	2x45	S74	3x29	T110	4x46	I154	5x44	S204	6x32	K270	7x38	N312
1x34	G35	2x46	L75	3x30	S111	4x47	L155	5x45	I205	6x33	A271	7x39	W313
1x35	M36	2x47	A76	3x31	I112	4x48	M156	5x46	V206	6x34	L272	7x40	I314
1x36	G37	2x48	C77	3x32	D113	4x49	V157	5x461	S207	6x35	K273	7x41	G315
1x37	I38	2x49	A78	3x33	V114	4x50	W158	5x47	F208	6x36	T274	7x42	Y316
1x38	V39	2x50	D79	3x34	L115	4x51	I159	5x48	Y209	6x37	L275	7x43	V317
1x39	M40	2x51	L80	3x35	C116	4x52	V160	5x49	V210	6x38	G276	7x45	N318
1x40	S41	2x52	V81	3x36	V117	4x53	S161	5x50	P211	6x39	I277	7x46	S319
1x41	L42	2x53	M82	3x37	T118	4x54	G162	5x51	L212	6x40	I278	7x47	G320
1x42	I43	2x54	G83	3x38	A119	4x55	L163	5x52	V213	6x41	M279	7x48	F321
1x43	V44	2x55	L84	3x39	S120	4x56	T164	5x53	I214	6x42	G280	7x49	N322
1x44	L45	2x551	A85	3x40	I121	4x57	S165	5x54	M215	6x43	T281	7x50	P323
1x45	A46	2x56	V86	3x41	E122	4x58	F166	5x55	V216	6x44	F282	7x51	L324
1x46	I47	2x57	V87	3x42	T123	4x59	L167	5x56	F217	6x45	T283	7x52	I325
1x47	V48	2x58	P88	3x43	L124	4x60	P168	5x57	V218	6x46	L284	7x53	Y326
1x48	F49	2x59	F89	3x44	C125	4x61	I169	5x58	Y219	6x47	C285	7x54	C327
1x49	G50	2x60	G90	3x45	V126	4x62	Q170	5x59	S220	6x48	W286	7x55	R328
1x50	N51	2x61	A91	3x46	I127	4x63	M171	5x60	R221	6x49	L287		
1x51	V52	2x62	A92	3x47	A128	4x64	H172	5x61	V222	6x50	P288		
1x52	L53	2x63	H93	3x48	V129			5x62	F223	6x51	F289		
1x53	V54	2x64	I94	3x49	D130			5x63	Q224	6x52	F290		
1x54	I55	2x65	L95	3x50	R131			5x64	E225	6x53	I291		
1x55	T56	2x66	M96	3x51	Y132			5x65	A226	6x54	V292		
1x56	A57	2x67	K97	3x52	F133			5x66	K227	6x55	N293		
1x57	I58			3x53	A134			5x67	R228	6x56	I294		
1x58	A59			3x54	I135			5x68	Q229	6x57	V295		
1x59	K60			3x55	T136			5x69	L230	6x58	H296		
1x60	F61			3x56	S137			5x70	Q231	6x59	V297		
								5x71	K232	6x60	I298		
								5x72	I233	6x61	Q299		
								5x73	D234				
								5x74	K235				
								5x75	S236				
								5x76	E237				

**ICL1**		**ECL1**		**ICL2**		**ECL2**						**H8**	
12x48	E62	23x49	M98	34x50	P138	45x50	C191					8x47	S329
12x49	R63	23x50	W99	34x51	F139	45x51	D192					8x48	P330
12x50	L64	23x51	T100	34x52	K140	45x52	F193					8x49	D331
12x51	Q65	23x52	F101	34x53	Y141							8x50	F332
				34x54	Q142							8x51	R333
				34x55	S143							8x52	I334
				34x56	L144							8x53	A335
				34x57	L145							8x54	F336
												8x55	Q337
												8x56	E338
												8x57	L339
												8x58	L340
												8x59	C341

Table S2: Sodium ions entries within 5 Å cutoff from D113^{3x32}. Each row corresponds to a single Na^+ ion observed in a specific replica and pH condition, with the ion identified by its atom ID and residue ID as in the reference structure protein_na.pdb (available in the Zotero repository). "Entry time" refers to the first frame in which the ion is observed within the cutoff, and "Length" indicates the total duration (in ns) the ion remained within 5 Å.

pH	Replica ID	Ion Atom	Ion Residue	Entry time [ns]	Length [ns]
6	1	5297	184	983.7	16.4
6	4	5303	190	366.9	0.7
6	4	5323	210	51.5	1.1
6	4	5330	217	13.1	4.7
7	1	5296	183	333.6	1.8
7	1	5323	210	431.7	84.9
7	2	5330	217	97.6	826.6
7	3	5305	192	67.4	482
7	4	5294	181	221.8	15.2
7	5	5290	177	241.7	4.9
8	1	5279	166	483.4	484
8	1	5291	178	79.3	118.1
8	1	5331	218	307.5	82.6
8	2	5295	182	61.8	920.7
8	3	5281	168	576.7	6.1
8	3	5300	187	140.1	102.7
8	3	5303	190	568.7	0.8
8	3	5304	191	675.5	2.6
8	3	5307	194	468.4	0.1
8	3	5308	195	251.2	2
8	3	5315	202	891.6	0.1
8	3	5317	204	698.8	21.7
8	3	5332	219	554.3	2.7
8	4	5317	204	535.1	431.3
8	5	5279	166	158.5	7.1
8	5	5282	169	743.6	1.3
8	5	5298	185	653.9	8.6
8	5	5299	186	417.1	4.4
8	5	5306	193	14.6	85.2
8	5	5310	197	189.2	22
8	5	5316	203	517.1	10.3
8	5	5320	207	566.3	5.8
8	5	5322	209	449.5	3.6
8	5	5325	212	489.6	3.3
9	1	5280	167	47.9	859.4
9	2	5284	171	267.0	10.5
9	2	5299	186	891.5	83.3
9	2	5302	189	418.2	106.3
9	2	5303	190	77.6	58.5
9	2	5308	195	622.6	169.6
9	2	5309	196	366.8	1.3
9	2	5314	201	156.2	82.8
9	2	5317	204	310.5	23.6
9	3	5281	168	917.0	7.8
9	3	5306	193	6.0	739.4
9	3	5312	199	938.2	47.8
9	4	5279	166	391.7	33.4
9	4	5282	169	290.6	6
9	4	5294	181	677.3	30.5
9	4	5307	194	925.8	4.4
9	4	5321	208	333.8	35.3
9	4	5324	211	750.6	97.5
9	4	5327	214	441.1	101.8
9	5	5316	203	112.2	519.1
9	5	5332	219	91.7	0.5

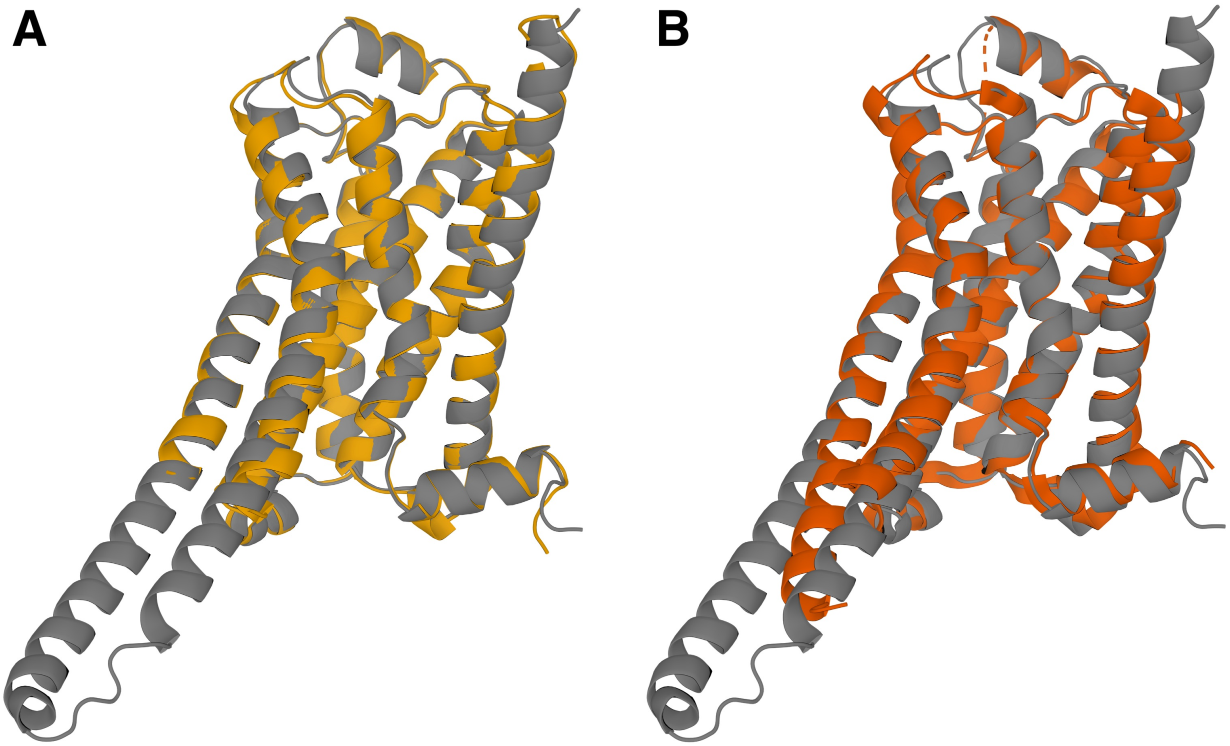

Figure S1: Comparison of the AlphaFold-reconstructed model and two experimental structures. A) Superposition between the model (gray) and the active (nanobody-bound) structure 3P0G (yellow). B) Superposition between the model (gray) and the active (G protein-bound) structure 3SN6 (orange). The initial model was obtained having as input the sequence of β_2AR and the Nb80 nanobody (residues 2-122), which explains the closer similarity to the nanobody-bound one.

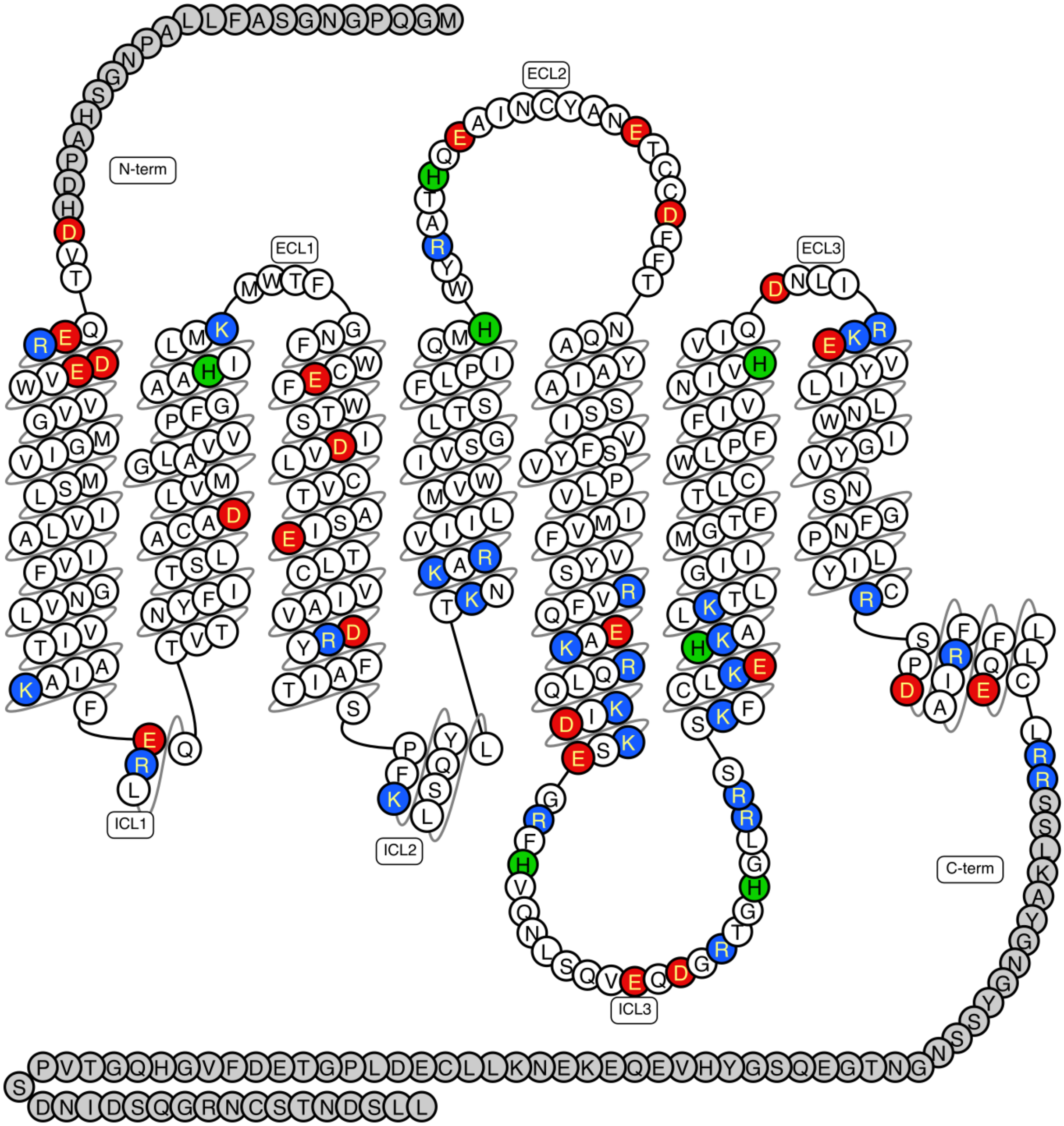

Figure S2: Snake diagram of the titrable residues in human β_2-adrenoceptor. The residues colored in red are the acidic residues, the blue ones are the basic ones, and the green one are histidines. The gray residues are the ones not included in our model. The snake diagram was obtained from the GPCRdb.[2]

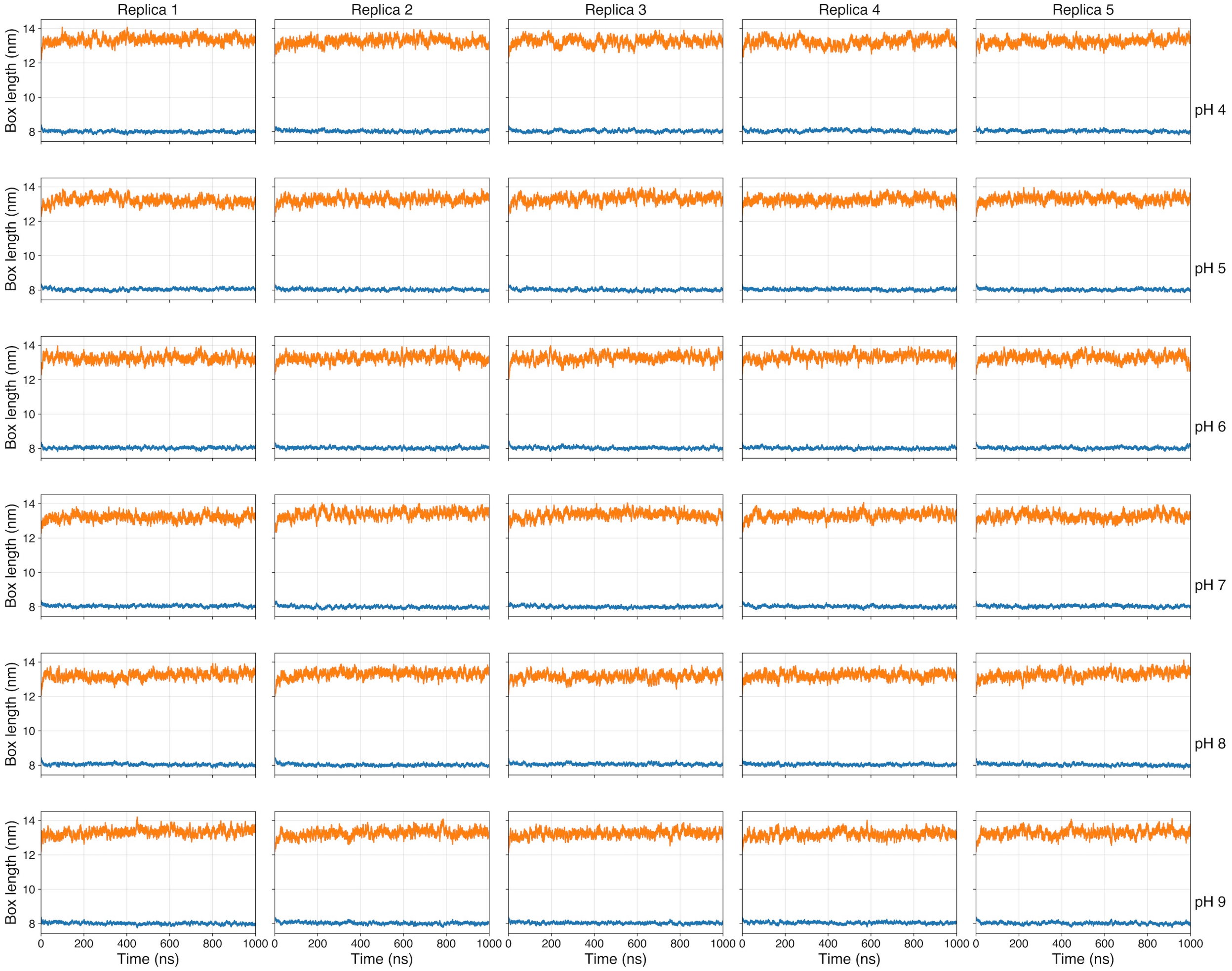

Figure S3: Time evolution of the simulation box dimensions under semi-isotropic pressure coupling. Each subplot corresponds to a specific pH condition (rows, pH 4-9) and replica (columns 1-5). Within each trajectory, the lateral box length (Box-X, which is equal to Box-Y, shown in blue) and the normal box length (Box-Z, shown in orange) are plotted as a function of simulation time. All the simulations show an equilibrium condition.

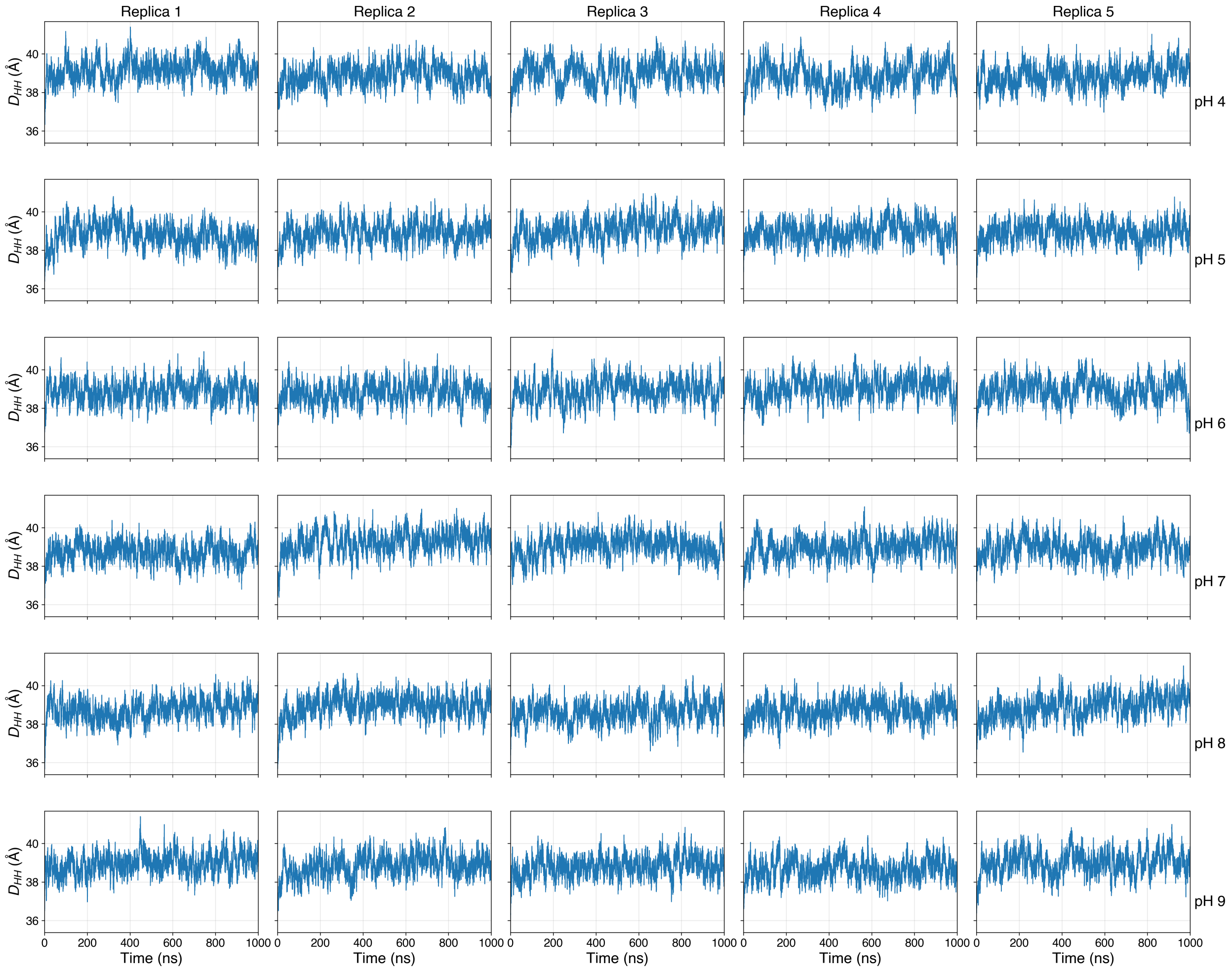

Figure S4: Time evolution of membrane thickness across pH and replica conditions. Each subplot shows the head-head distance D_{HH} (computed as the z-distance between the phosphate group of the upper and lower layer) in Å as a function of simulation time. Rows correspond to pH values from 4 (top) to 9 (bottom), and columns correspond to the five independent replicas (Rep 1-5). Membrane thickness was calculated using the LeafletFinder module in MDAnalysis.

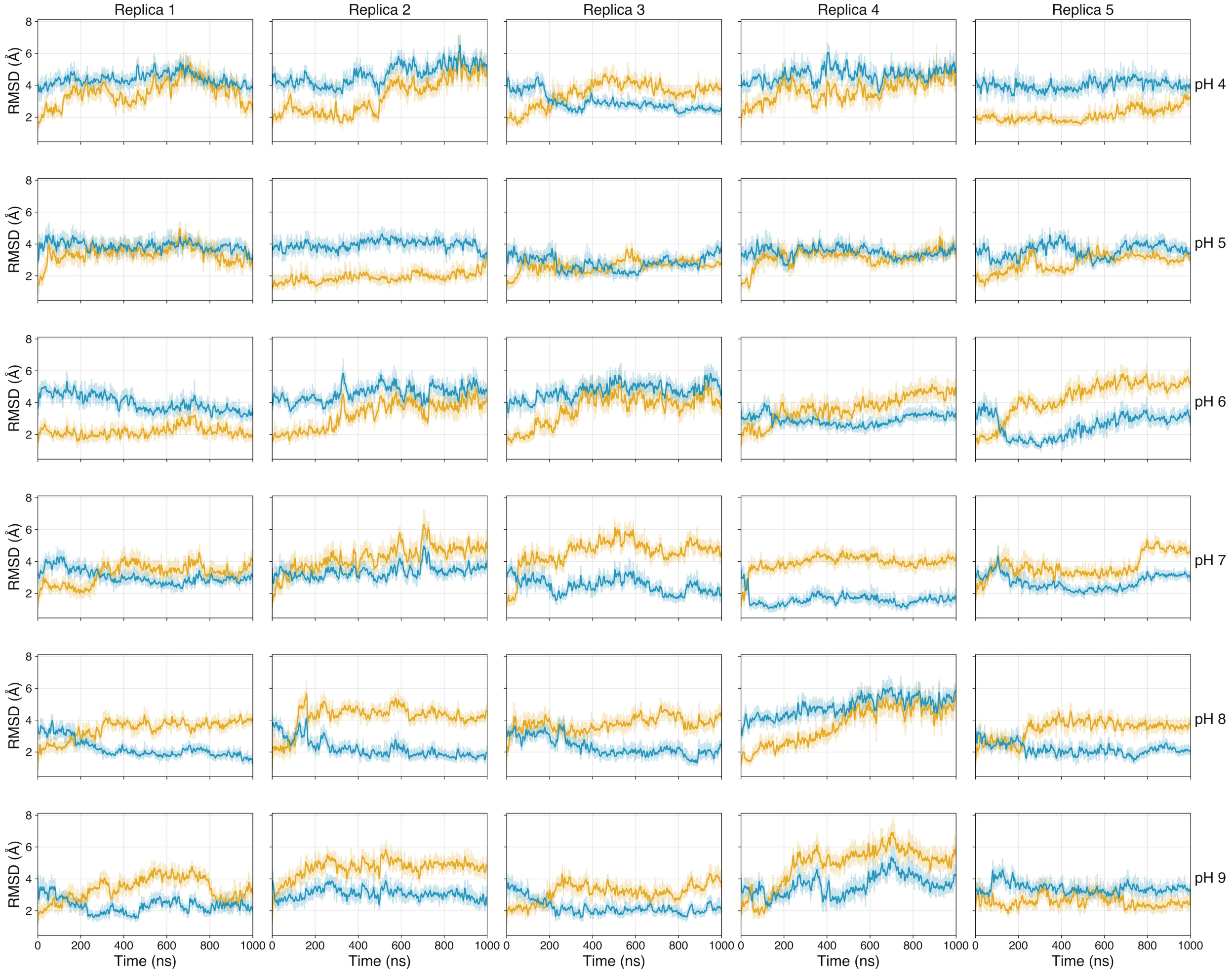

Figure S5: Root Mean Square Deviation (RMSD) of transmembrane regions across pH and replica conditions relative to active and inactive reference structures. Each subplot shows the time evolution of the RMSD values (in Å) computed for each replica (columns 1–5) at a specific pH (rows, from pH 4 at the top to pH 9 at the bottom). RMSD was calculated using GROMACS[3] over five independent molecular dynamics trajectories per pH condition. RMSD reflects the structural deviation of the receptor from two reference states: the inactive structure (PDB ID: 2RH1) shown in blue, and the active structure (PDB ID: 3P0G) shown in yellow. For each trajectory, only the Cα atoms of TM3 (residues 102-137), TM5 (196-227), and TM6 (266-299) were used for RMSD calculation, after a least-squares alignment to the Cα atoms of TM1 (29-61), TM2 (66-97), TM4 (146-172), and TM7 (304-328). The analysis was restricted to residues in common in both 3P0G and 2RH1 due to missing residues in the reference structures. Raw RMSD traces are shown in light colors, while the overlaid darker lines represent Savitzky-Golay-smoothed data for visual clarity.

		Ionic lock distance	Y-Y distance	NPxxY RMSD	PIF RMSD
pH 4	replica 1	✗	✗	✗	✗
	replica 2	✗	✗	✗	✗
	replica 3	✓	✓	⚠	✗
	replica 4	✗	✗	✗	✗
	replica 5	✗	✓	✓	⚠
pH 5	replica 1	✗	✗	✗	⚠
	replica 2	✗	✗	⚠	⚠
	replica 3	✓	⚠	⚠	⚠
	replica 4	✗	✗	✗	✗
	replica 5	⚠	✗	✗	⚠
pH 6	replica 1	✗	✗	✗	✗
	replica 2	✗	⚠	✗	✗
	replica 3	✗	⚠	✗	✗
	replica 4	✓	✓	✗	✗
	replica 5	✓	✓	✓	✓
pH 7	replica 1	✓	✗	✗	⚠
	replica 2	✓	✗	✗	⚠
	replica 3	✓	⚠	✓	✓
	replica 4	✓	✓	✓	✓
	replica 5	✓	⚠	⚠	✓
pH 8	replica 1	✓	⚠	⚠	✓
	replica 2	✓	⚠	✓	✓
	replica 3	✓	⚠	✓	✓
	replica 4	✗	⚠	✗	✗
	replica 5	✓	⚠	✗	✓
pH 9	replica 1	✓	⚠	⚠	✓
	replica 2	✓	⚠	⚠	✓
	replica 3	✓	⚠	⚠	✓
	replica 4	✓	⚠	⚠	⚠
	replica 5	✗	✗	⚠	⚠

✗: Remains active ⚠: Partially inactivated ✓: Inactivated

Figure S6: Table of the microswitches inactivation during the performed simulations.

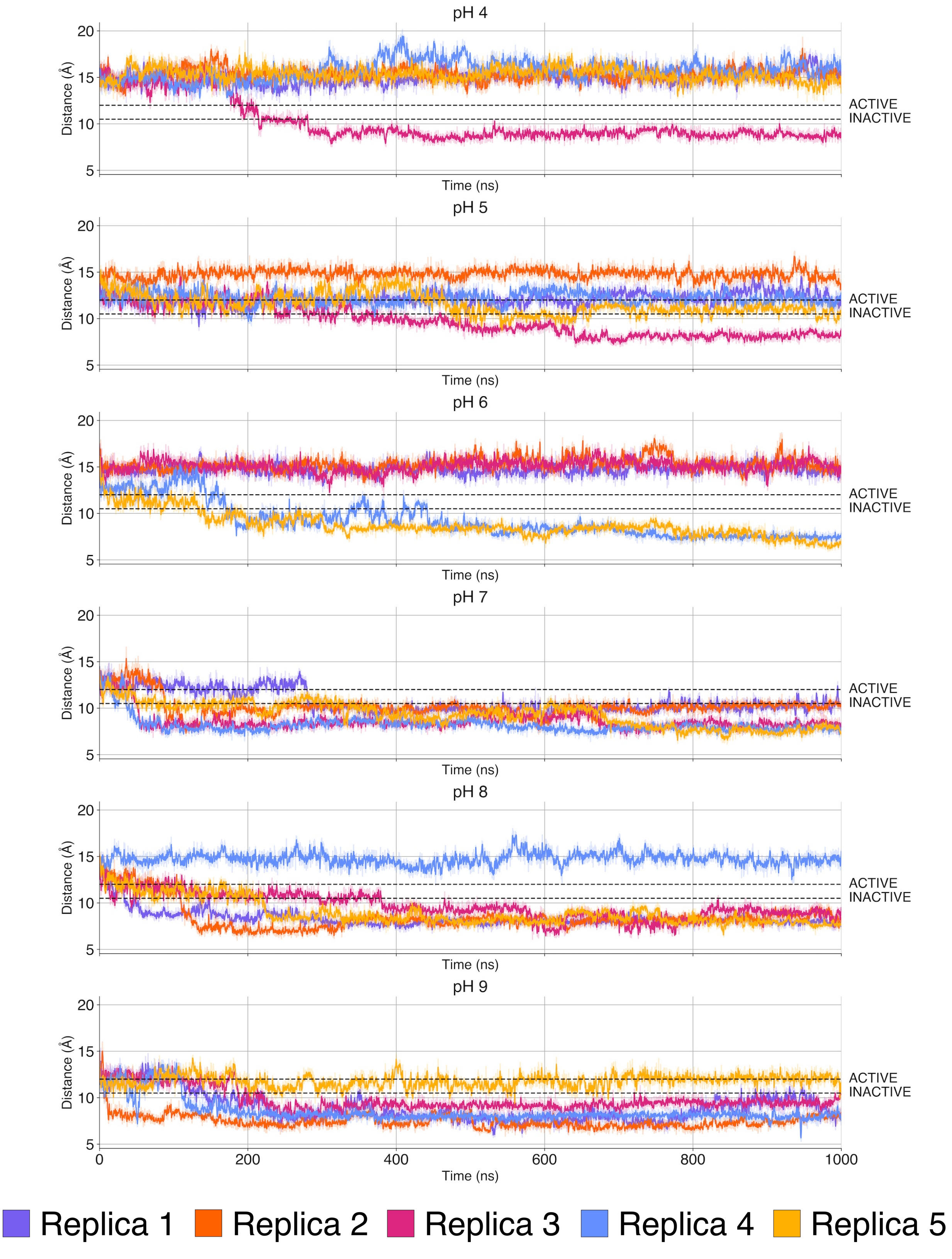

Figure S7: Time evolution for the Ionic lock distance in all the simulations performed.

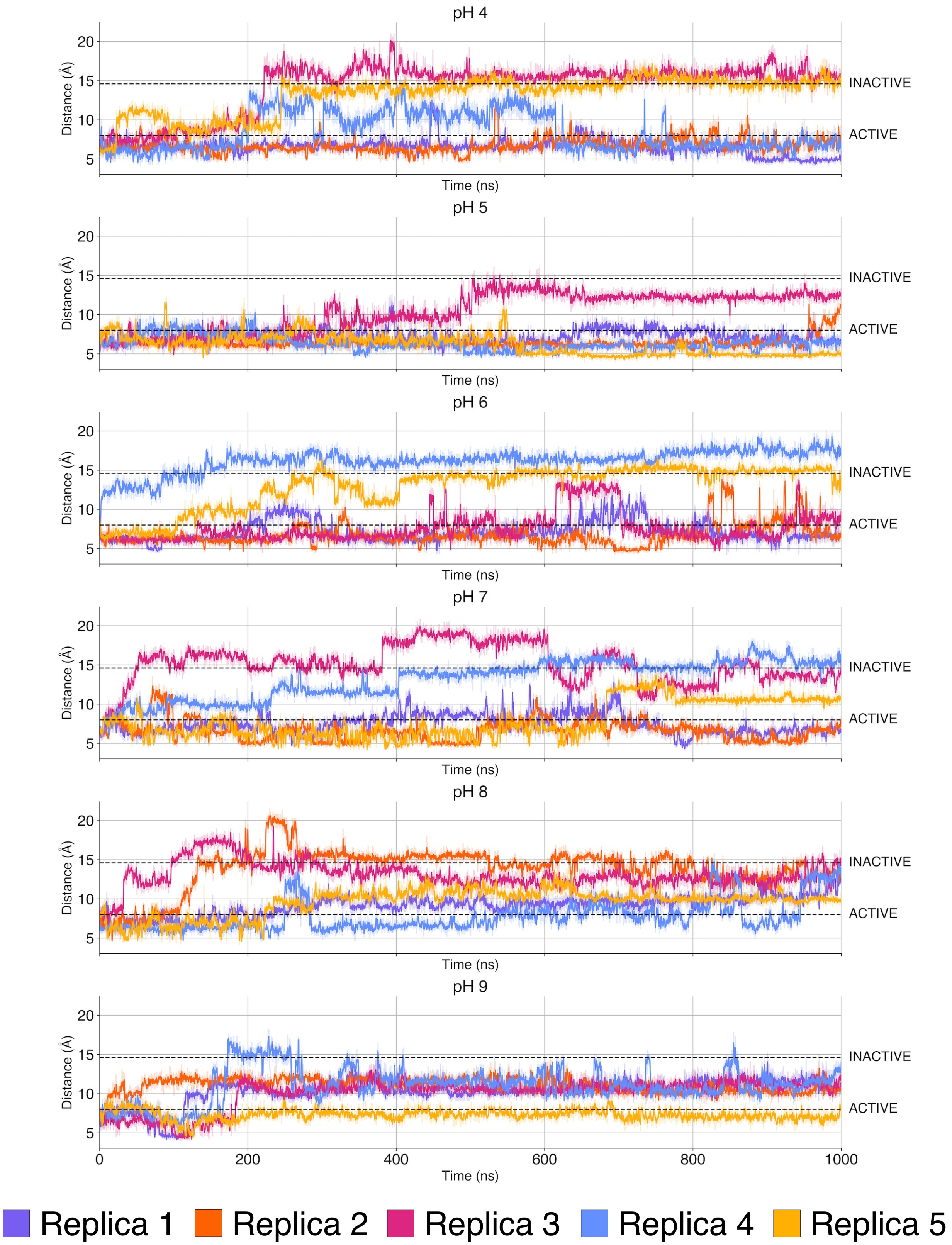

Figure S8: Time evolution for the Y-Y distance in all the simulations performed.

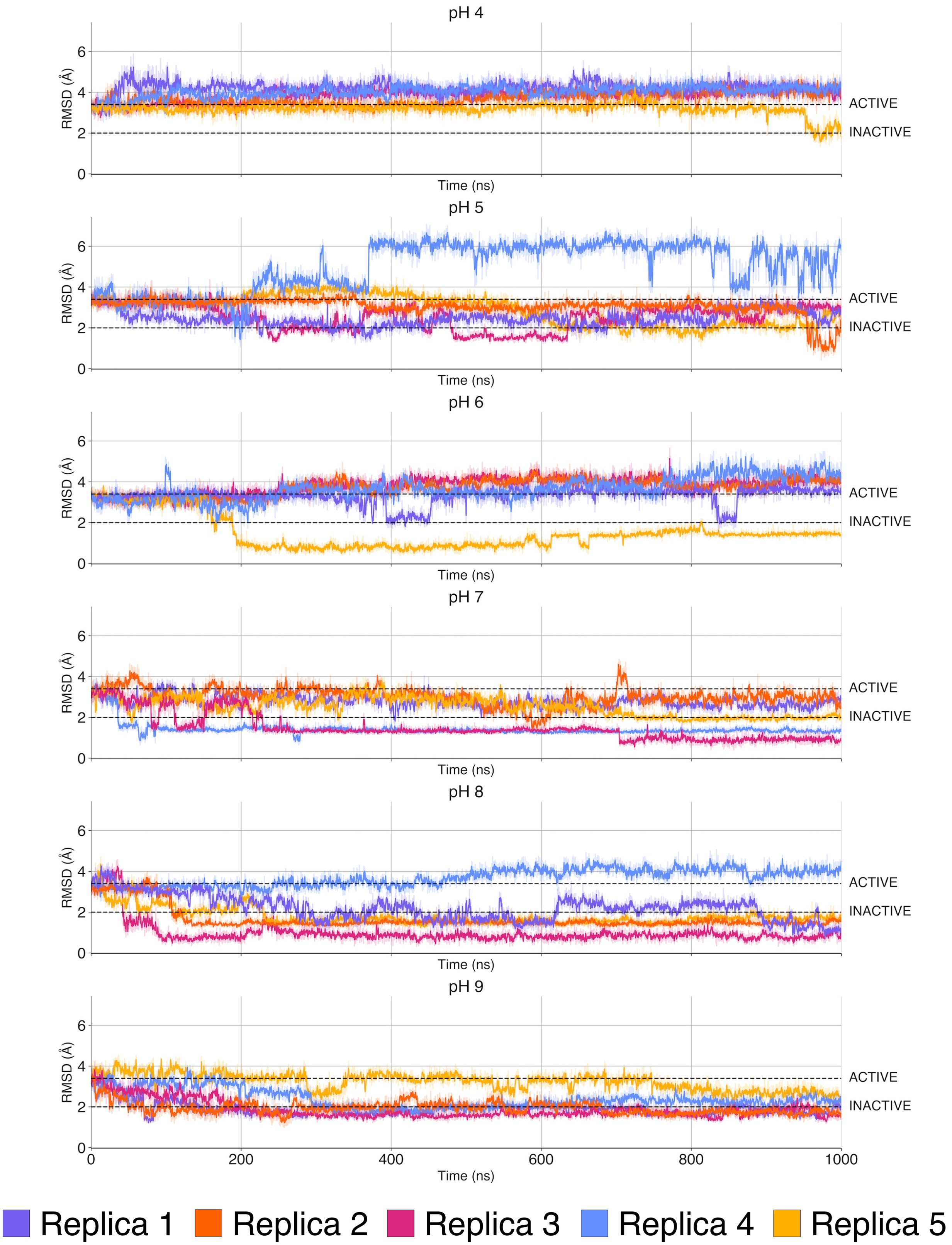

Figure S9: Time evolution for the PIF motif RMSD in all the simulations performed.

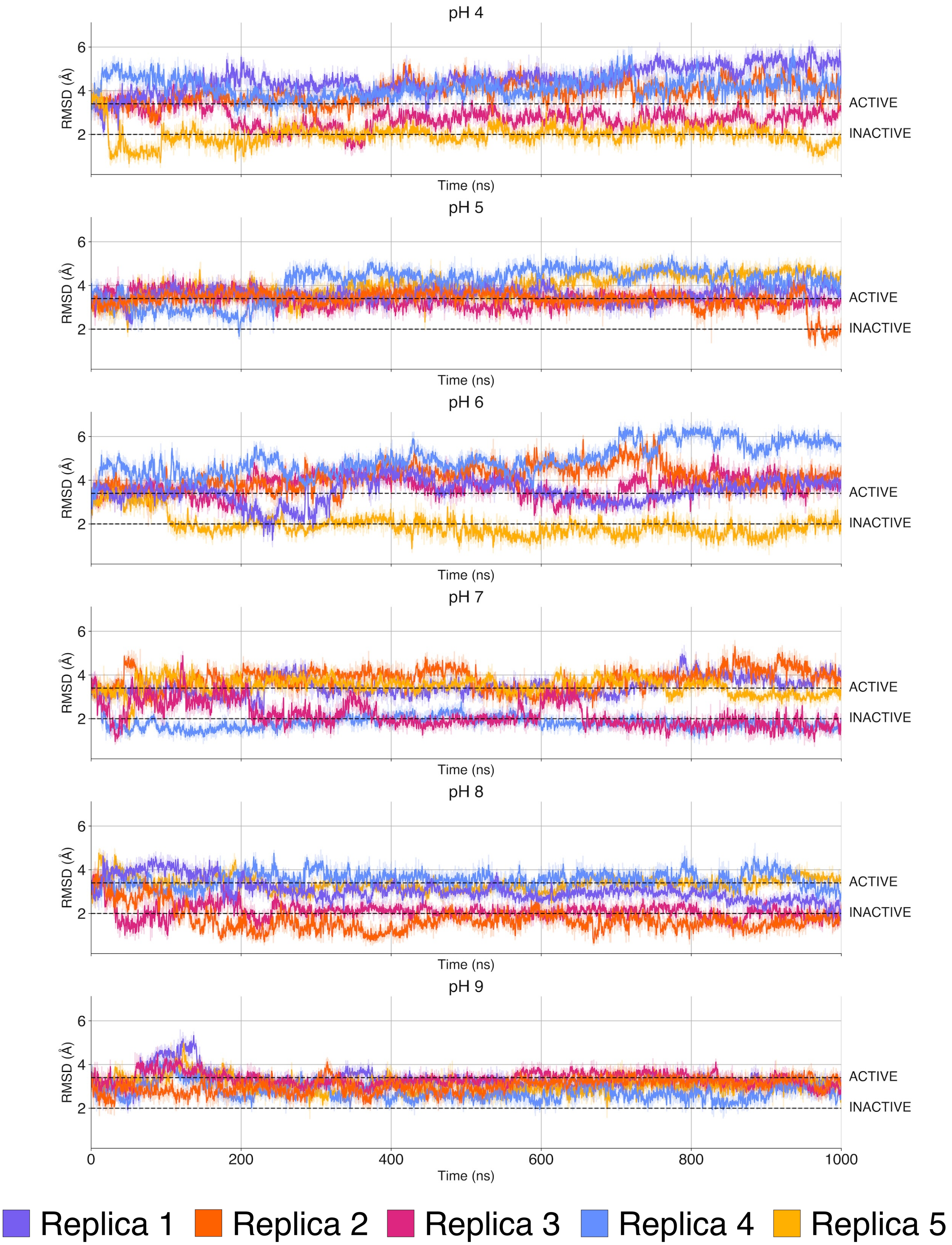

Figure S10: Time evolution for the NPxxY motif RMSD in all the simulations performed.

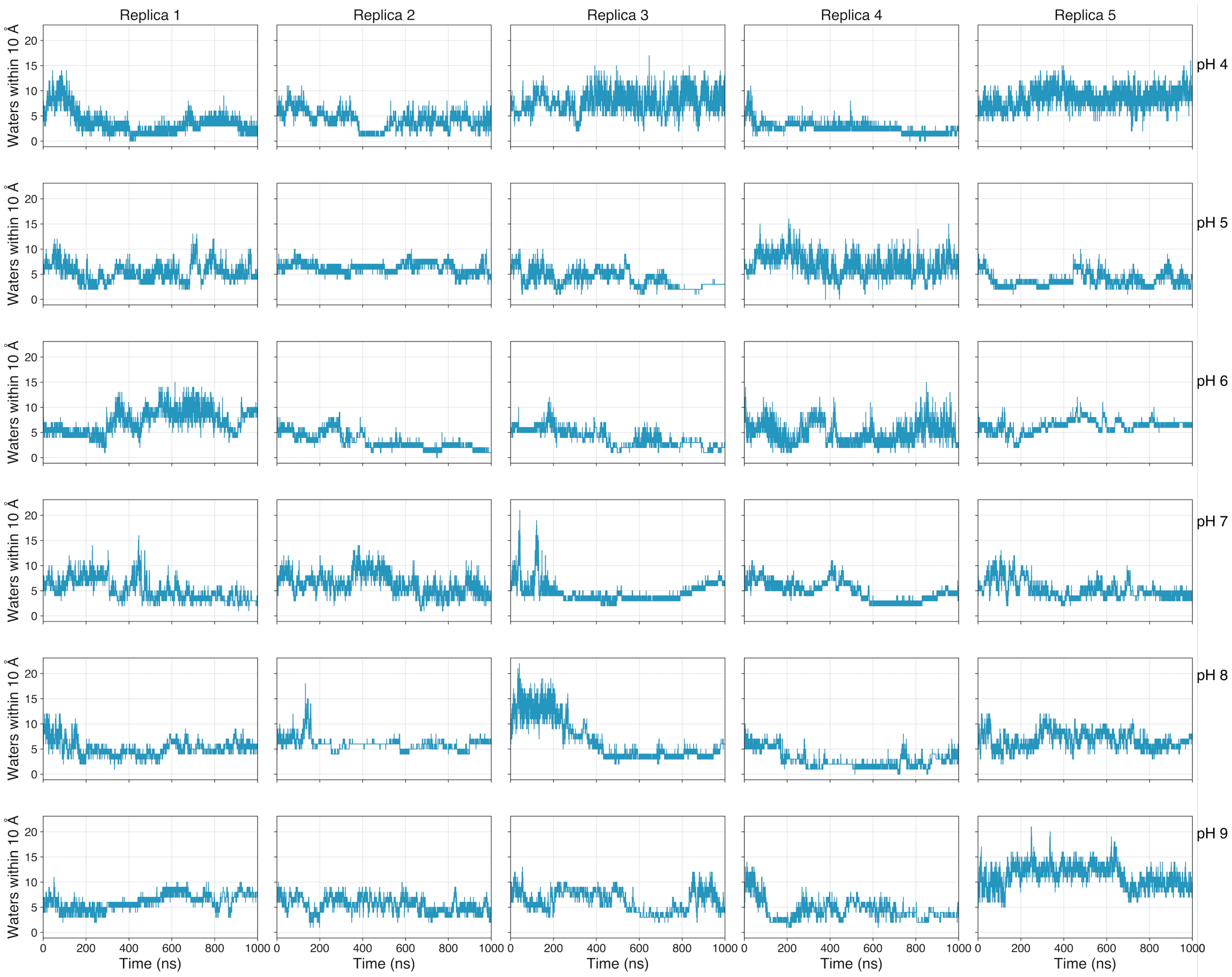

Figure S11: Number of water molecules within 10 Å of D79^{2x50} as a function of time. Each subplot corresponds to one of five replicas (columns) at a given pH (rows, pH 4-9). The analysis was performed on all the 30 trajectories. A water molecules is considered if its oxygen atom has a distance from C_γ of D79^{2x50} smaller than the threshold. Hydration counts are descriptive and do not imply deprotonation propensity. Across pH 4–9 the pocket remains partially dehydrated ($\sim$6 waters) and D79^{2x50} stays neutral in our λ-dynamics CpHMD; inactivation is therefore not contingent on D79^{2x50} deprotonation.

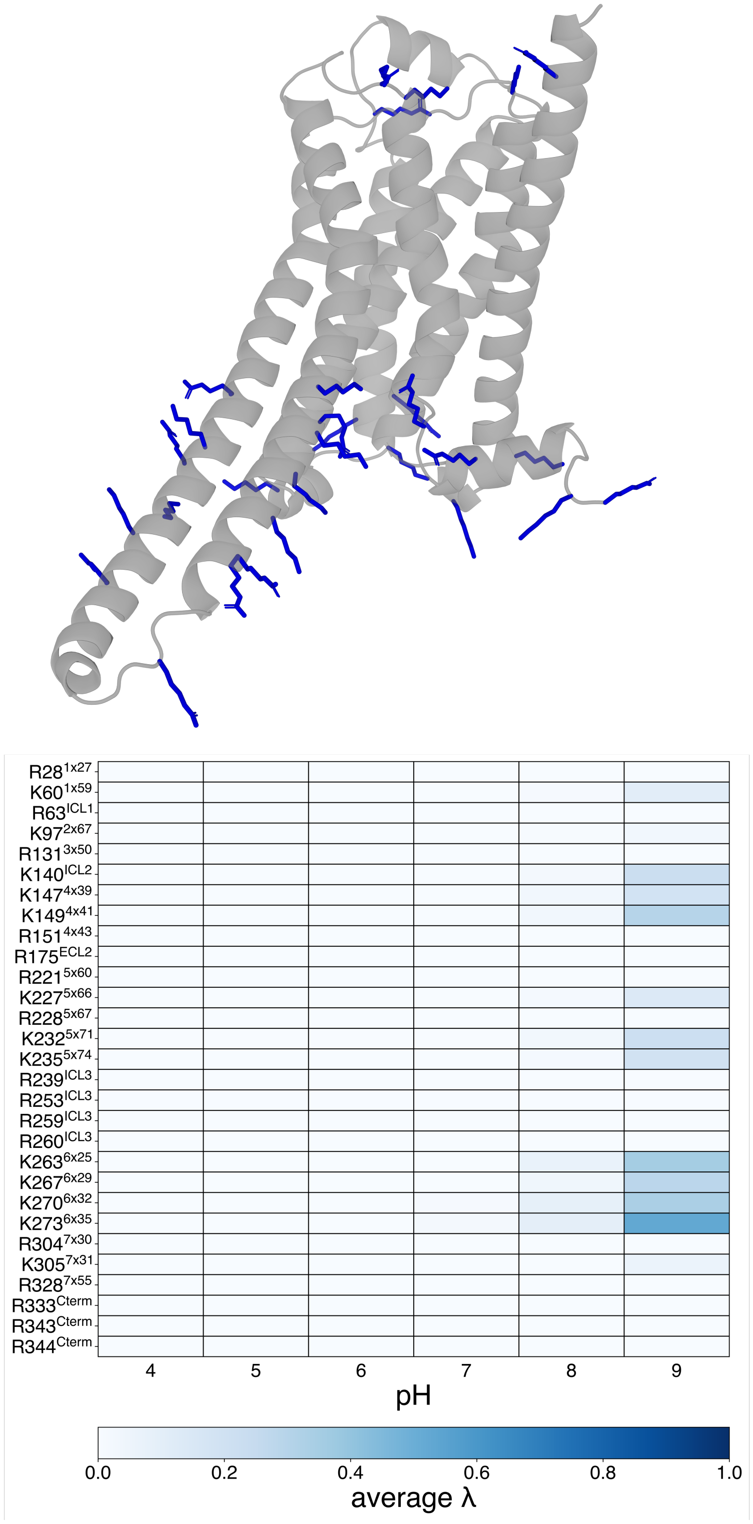

Figure S12: Titrable basic residues and their protonation state at different pH. On top, the structure of β_2AR with basic residues highlighted in blue. Below, a colormap representing the charge state of such residues. All the basic amino acids are neutral for all the simulation from pH 4 to 8. At pH 9 a small number of residues show a partial change in protonation state.

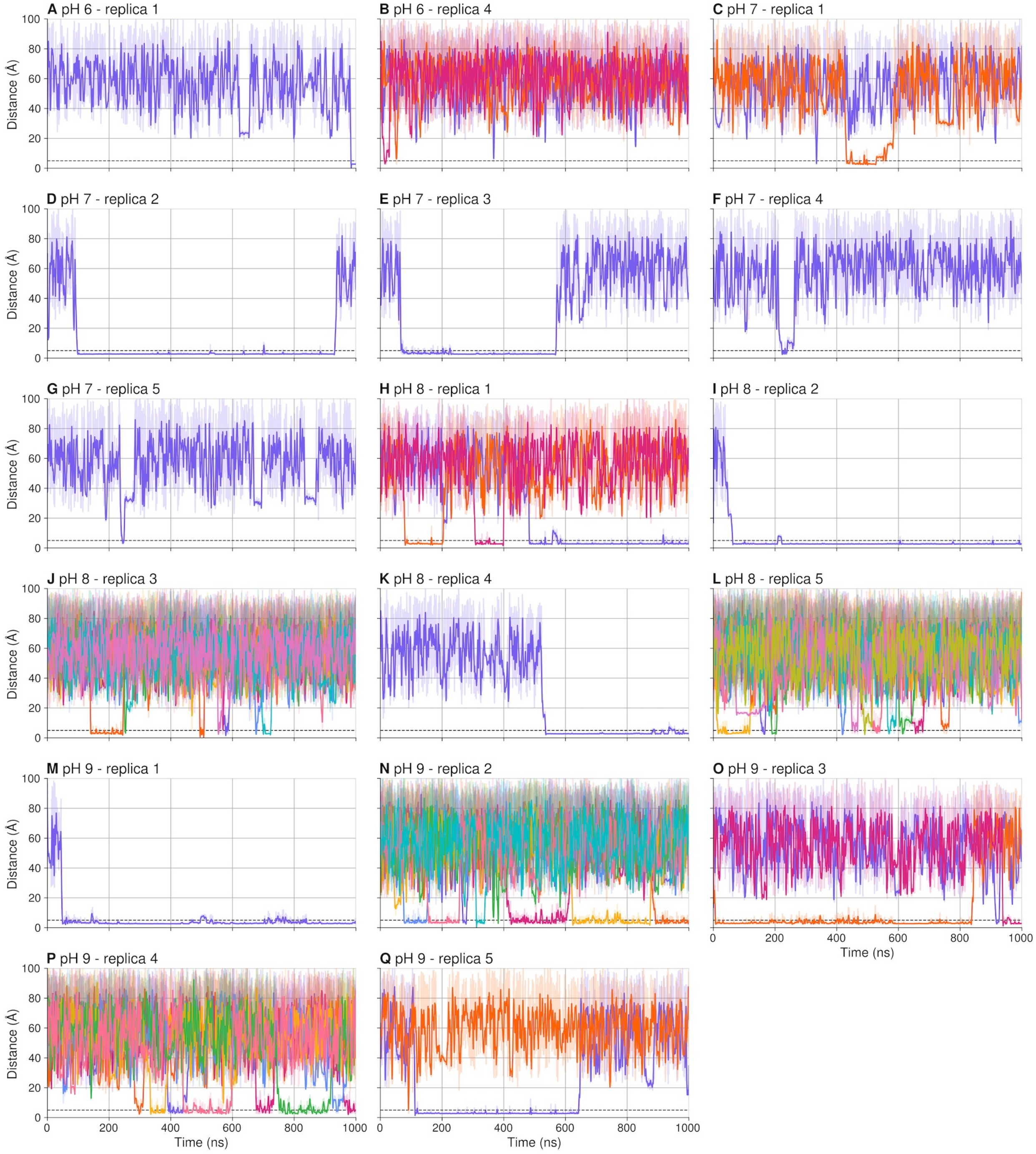

Figure S13: Time evolution of distances between Na^+ ions and the Cγ atom of residue D113^{3x32} across different pH conditions and replicas. Each subplot corresponds to a specific pH and replica, with individual ion traces shown. Raw distance data (semi-transparent lines) are plotted alongside smoothed trajectories (solid lines) obtained using a Savitzky–Golay filter (window size = 51 frames, polynomial order = 3). The black dashed line marks the 5 Å distance threshold used to define ion-residue contact. Distances were computed from molecular dynamics trajectories using MDAnalysis,[4] and time is shown in nanoseconds.

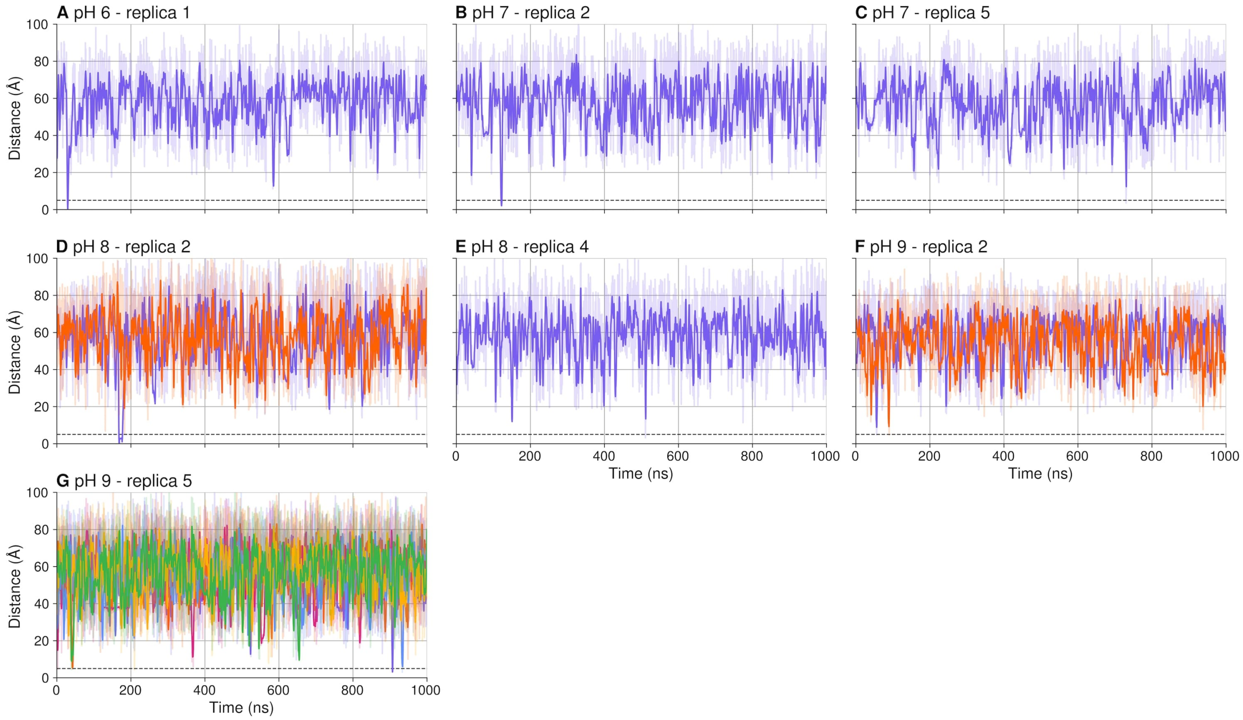

Figure S14: Time evolution of distances between Na^+ ions and the CG atom of residue D130^{3x49} across different pH conditions and replicas. Each subplot corresponds to a specific pH and replica, with individual ion traces shown. Raw distance data (semi-transparent lines) are plotted alongside smoothed trajectories (solid lines) obtained using a Savitzky–Golay filter (window size = 51 frames, polynomial order = 3). The black dashed line marks the 5 Å distance threshold used to define ion-residue contact. Distances were computed from molecular dynamics trajectories using MDAnalysis,[4] and time is shown in nanoseconds.

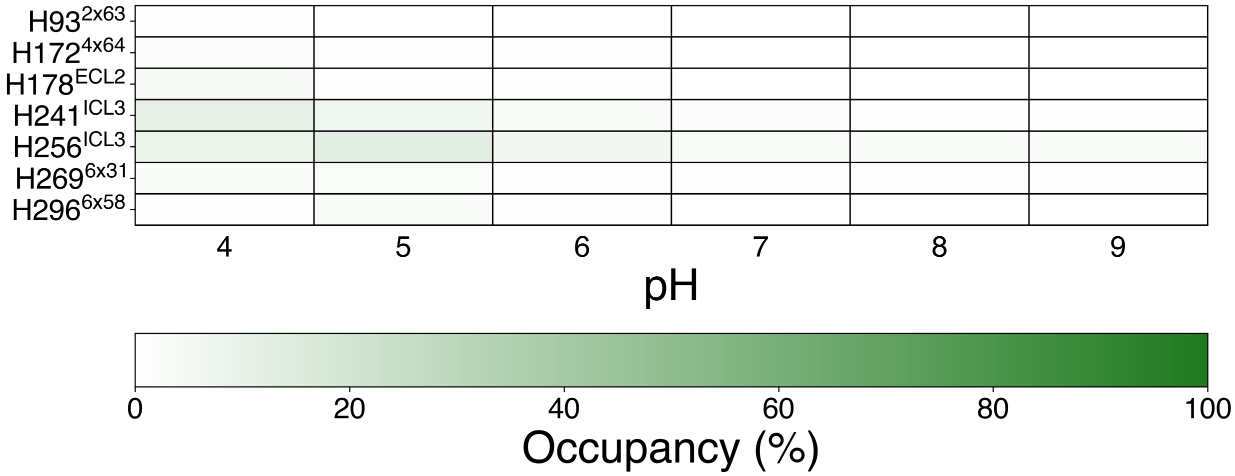

Figure S15: Occupancy of Cl^- ions to histidine residues. In all the pH conditions studied here we do not see a lasting binding between chlorine and charged histidine residue: the only cases where the time percentage overcomes 10% is for pH between 4 and 5, with residues in the ICL3 completely exposed to solvent (H241^{ICL3} and H256^{ICL3}).

References

(1) Gowers, R.; Linke, M.; Barnoud, J.; Reddy, T.; Melo, M.; Seyler, S.; Domański, J.; Dotson, D.; Buchoux, S.; Kenney, I.; Beckstein, O. MDAnalysis: A Python Package for the Rapid Analysis of Molecular Dynamics Simulations. Proceedings of the 15th Python in Science Conference. 2016; p 98–105.

(2) Herrera, L. P. T.; Andreassen, S. N.; Caroli, J.; Rodríguez-Espigares, I.; Kermani, A. A.; Keserű, G. M.; Kooistra, A. J.; Pándy-Szekeres, G.; Gloriam, D. E. GPCRdb in 2025: adding odorant receptors, data mapper, structure similarity search and models of physiological ligand complexes. *Nucleic Acids Research* **2024**,

(3) Jansen, A.; Aho, N.; Groenhof, G.; Buslaev, P.; Hess, B. phbuilder: A Tool for Efficiently Setting up Constant pH Molecular Dynamics Simulations in GROMACS. *Journal of Chemical Information and Modeling* **2024**, *64*, 567–574.

(4) Richard J. Gowers; Max Linke; Jonathan Barnoud; Tyler J. E. Reddy; Manuel N. Melo; Sean L. Seyler; Jan Domański; David L. Dotson; Sébastien Buchoux; Ian M. Kenney; Oliver Beckstein MDAnalysis: A Python Package for the Rapid Analysis of Molecular Dynamics Simulations. Proceedings of the 15th Python in Science Conference. 2016; pp 98 – 105.
